# Dataset of full-length transcriptome assembly and annotation of *apocynum venetum* using pacbio sequel II

**DOI:** 10.1016/j.dib.2020.106494

**Published:** 2020-11-04

**Authors:** Tingting Zhang, Mao Li, Ya Guang Zhan, Gui Zhi Fan

**Affiliations:** Key Laboratory of Saline-alkali Vegetation Ecology Restoration, Ministry of Education, Northeast Forestry University, Harbin 150040, China

**Keywords:** *Apocynum venetum*, Transcriptomics, Pacbio sequel ii, Functional annotation

## Abstract

*Apocynum venetum*, which belongs to Apocynaceae, is widely distributed throughout salt-barren zones, desert steppes, and alluvial flats of the Mediterranean area and Northwestern China. *Apocynum venetum* has long been used in traditional Chinese medicine because of its anti-inflammation, anti-oxidative, anti-hypertensive, anti-cancer, and bactericidal effects. However, the absence of genetic information on *Apocynum venetum* is an obstacle to understanding its stress resistance or medicinal function. This work was aimed at generating a full-length transcriptome of *Apocynum venetum* using Pacific Bioscience (PacBio) Single Molecule Real-Time (SMRT) sequencing technology. A total of 18,524 unigenes were obtained, and 18,136 unigenes were successfully annotated. The raw data were uploaded to SRA database, and the BioProject ID is PRJNA650225. The above data will provide the basis for further exploration and understanding of the molecular mechanism in stress resistance or medicinal function of *Apocynum venetum*.

## Specifications Table

**Table:** 

Subject	Plant Science
Specific subject area	Full-length Transcriptomics
Type of data	Table, Figure
How data were acquired	PacBio Sequel II sequencing platform
Data format	Raw, Analyzed, Filtered
Parameters for data collection	Total RNA was extracted from 30-day-old *Apocynum venetum* plantlets to construct cDNA library. Full-length transcriptome sequences of *Apocynum venetum* was obtained using PacBio SMRT sequencing technology. The unigene dataset was functionally annotated using Gene Ontology (GO), Eukaryotic Orthologous Groups of proteins (KOG), Kyoto Encyclopedia of Genes and Genomes (KEGG), Swiss-Prot Protein Database (Swiss-Prot), and NCBI Non-Redundant Proteins (NR).
Description of data collection	After preprocessing and assembling clean subthreads (47.4 GB, 35,351,576 subreads), a total of 482,757 full-length reads and 472,648 full-length non-chimeric reads were obtained. CD-HIT software is used to cluster and remove redundancy. 18,524 full-length single genes were obtained, and 18,136 single genes were successfully annotated.
Data source location	Institution: Northeast Forestry University Country: China Latitude and longitude (and GPS coordinates) for collected samples/data: 47°50′36″ N, 88°08′04″ E
Data accessibility	Raw RNA-Sequencing data and assembly file are available from the Sequence Read Archive (SRA) on NCBI, data identification number PRJNA650225. (https://www.ncbi.nlm.nih.gov/bioproject/PRJNA650225/). The associated annotation data are available as Supplementary Material.

## Value of the Data

• The full-length transcriptome data of *Apocynum venetum* using Single Molecule Real-Time (SMRT) sequencing technology provide an important reference for the scientific community to understand of the molecular mechanism and physiological function of *Apocynum venetum*.
• Full-length transcripts will be useful for gene discovery, characterization and cloning.
• The data will be useful for the genetic improvement of *Apocynum venetum* or other plants.
• Researchers can use their own bioinformatics algorithms to further process and analyze the original sequence data.

## 1 Data Description

**Fig. 1:**
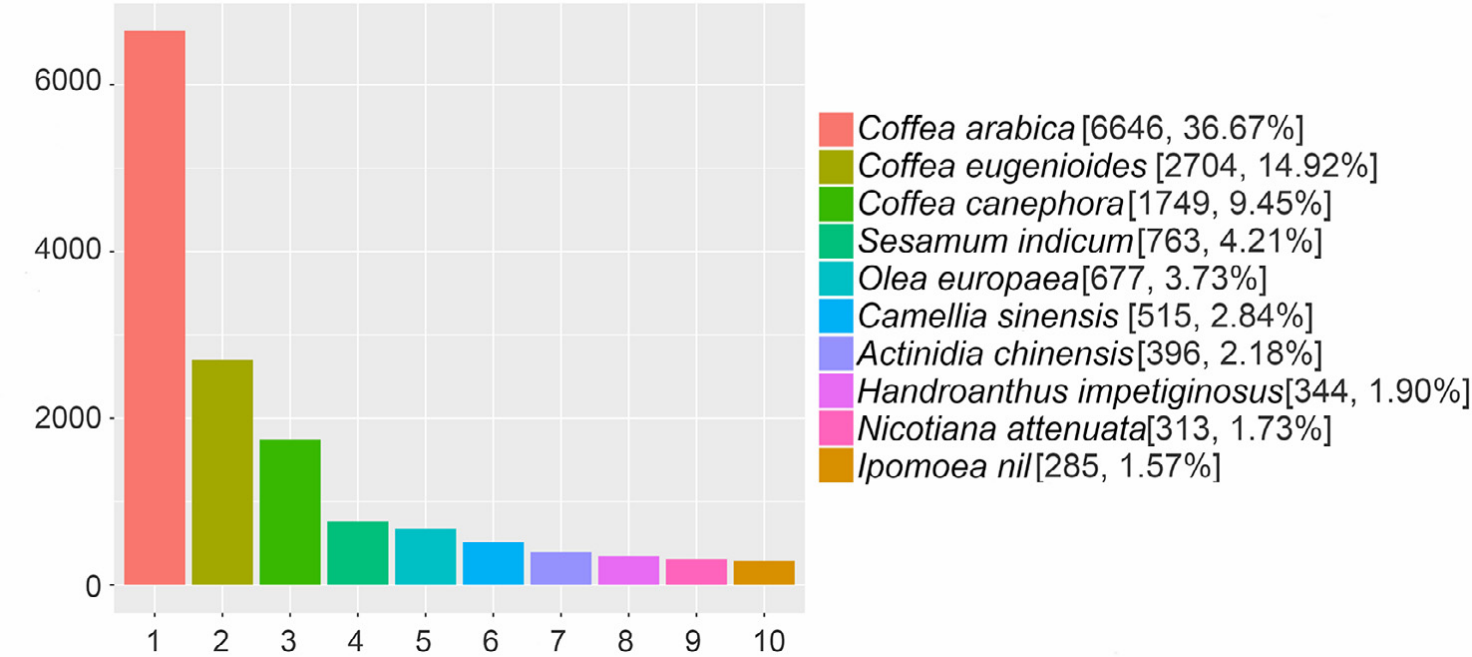
Distribution of homologous species in *Apocynum venetum* with NR annotation.

**Fig. 2:**
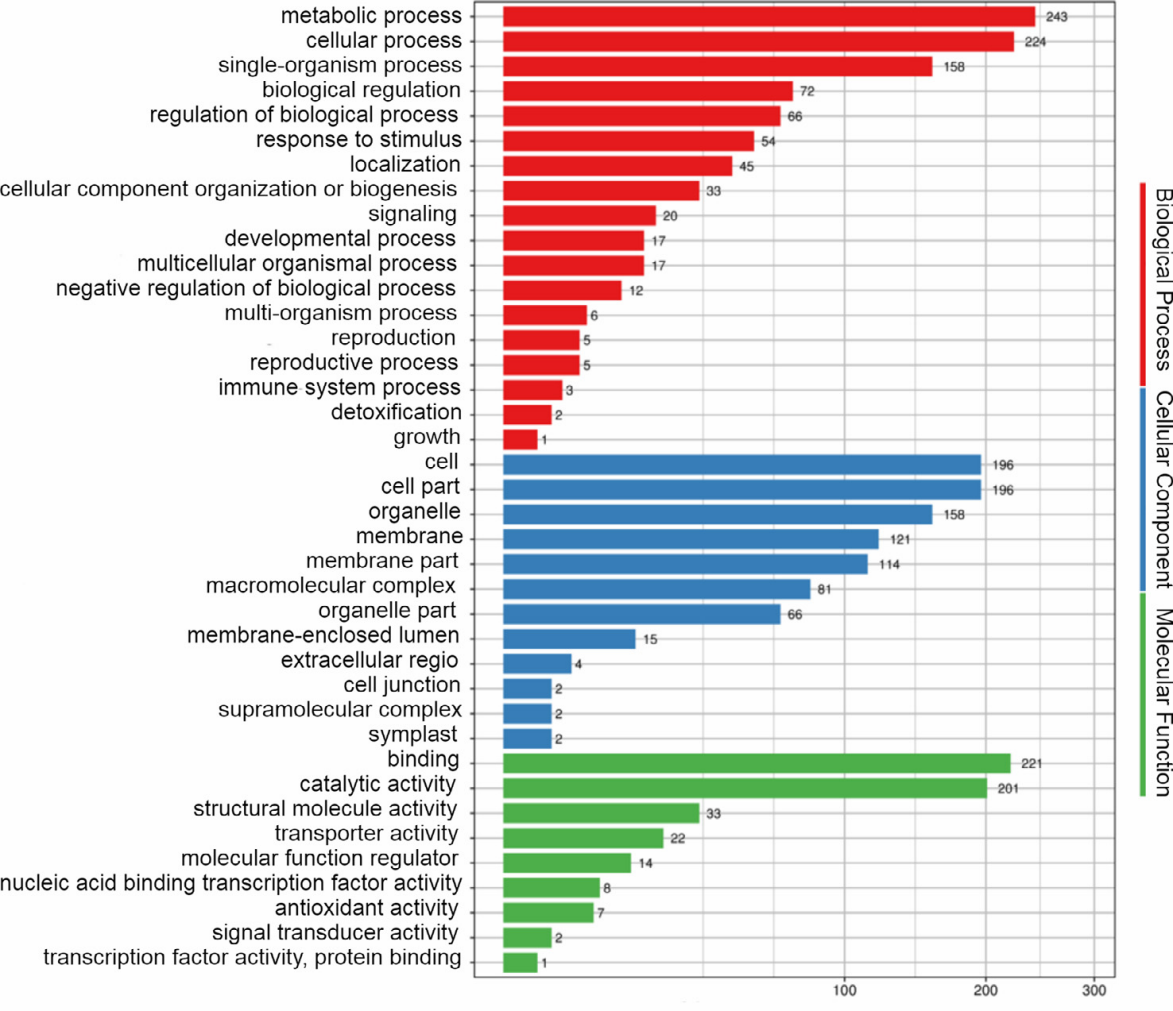
GO category distribution of Blast annotated *Apocynum venetum* unigenes.

Details of statistics of transcripts and unigene for the full-length transcriptome of *Apocynum venetum* were provided in Table 1. The sequencing results generated 47.4 GB (35,351,576 reads) of clean data, which had been deposited in the SRA database (PRJNA650225). A total of the 18,524 unigenes were sequenced, and 18,136 unigenes were successfully annotated using NR, GO, Swiss-Prot, KOG and KEGG databases (Table 2). Among of them, 18,126 full-length unigenes were annotated through the NR databases, and the highest homology ratio with Coffee arabic was 36.67% (Fig. 1 and Supplementary Table S1); 472 Unigenes were classified into 3 main GO categories (Biological Process, Cellular Component, Molecular Function) and 39 sub-categories (Fig. 2 and Supplementary Table S2); 13,717 in Swiss-Prot; 11,150 in KOG; and 8229 in KEGG (Supplementary Tables S3, S4 and S5). The classification of transcripts using the Protein Families (Pfam) database was shown in Supplementary Table S6.

**Table 1:** Statistics of transcripts and Unigene for the full-length transcriptome of *Apocynum venetum*.

	Transcript subreads	Circular consensus sequence(CCS) reads	Full-length non-chimericRead	Unigene
Total Number	35,351,576	577,261	472,648	18,524
Max Length	207,856	12,824	8394	7691
Min Length	51	106	63	194
N50 Length	1473	1704	1592	1838
Mean Length	1341	1536	1413	1654

**Table 2:** Statistics of annotated Unigene in *Apocynum venetum*.

	Annotated number
NR	18,126
Swissprot	13,717
KOG	11,150
KEGG	8229
GO	472
All annotated	18,136

## 2 Experimental Design, Materials and Methods

### 2.1 Sample collection

The seeds of *Apocynum venetum* were collected from Xinjiang Uygur Autonomous Region and identified by associate researcher Jiang Li. 30-day-old *Apocynum venetum* plantlets (10 plants) cultured on WPM medium were mixed for RNA extraction.

### 2.2 Total rna extraction and library construction

Total RNA was isolated from *Apocynum venetum* samples. The integrity, quality and concentration of RNA samples were checked by Agarose gel electrophoresis and Nanodrop 2000. The library was constructed after the samples were qualified. The main processes were as follows: 1) Total RNA was reversely transcribed into cDNA using a Clonetech SMARTerTM PCR cDNA Synthesis Kit that was optimized for preparing high-quality, full-length cDNAs. The 3′ terminal of eukaryotic mRNA had a Poly(A) tail structure. The primer A with Oligo dT was used for A-T base pairing with Poly(A) as the primer for reverse synthesis of cDNA. Add primer B to the terminal of the full-length cDNA synthesized in reverse. The obtained full-length cDNA was amplified by PCR. The amplified product was purified by PB magnetic beads and quantified by Qubit 2.0. 2) Use BluePippin to screen cDNA fragments above 4Kb. The amplified cDNA fragments were amplified by PCR again and the full-length cDNA was purified by PB magnetic beads. 3) Terminal-repair the full-length cDNA and connected the SMRT dumbbell adapter. 4) Used exonuclease to digest the fragments that were not connected to the jointer. Use PB magnetic beads for purification again to obtain a sequencing library. 5) After the library was constructed, Qubit 2.0 was used for accurate quantification. Then used Agilent 2100 to detected the library size. Sequencing was performed after the library size was qualified.

### 2.3 Transcriptome sequencing and assembly

Used the PacBio Sequel II sequence platform to sequence qualified libraries [1,2]. The subreads were acquired from raw sequencing reads using the SMRT Link (version7.0.0.63985; parameter -min_passes 3, -min_length 50, -max_length 15,000) pipeline supported by PacBio's official, and Circular Consensus Sequence (CCS) reads were extracted out of subreads’ BAM file. Through IsoSeq, CCS reads were classified into full-length (FL), full-length non-chimeric (FLNC), non-full-length (NFL) based on cDNA primers and Poly(A) tail signal. Subsequently, the FLNC reads were clustered by Iterative Clustering and Error correction (ICE) tool to generate the cluster consensus isoforms. Finally, the NFL sequence was used to modify the obtained consistent sequence (polished) to obtain high-quality sequence for subsequent analysis [3,4]. To yield a final set of non-redundant transcript sequences, CD-HIT (version4.8.1; parameter -c 0.95, -G 0, -aL 0.00, -aS 0.99, -AS 30) software was used to merge highly similar sequences and remove redundant sequences from high-quality transcript. Used Diamond (version0.9.24; parameter -more-sensitive, -k 10, -e le-5) to aligned the sequences to various databases. Got the protein with the highest sequence similarity and annotate the protein function information. TransDecoder (version 5.5.0; parameter -G universal, -S, -m 100) was used to identify the candidate Coding Sequence (CDS) regions within transcript sequences.

### 2.4 Functional annotation of full-length transcriptome sequences

NR [5] database was used to homology searches (E-value = 1e−5). GO [6] and KOG [7,8] was used to annotations gene function. The Swiss-Prot [9] and Pfam [10] was used to classifies transcript. The KEGG [11] tuning parameter -species ko; -e le-5 was used to compare and annotate transcripts.

## 3 Funding

This work was supported by the Foundation of State Key Laboratory of Desert and Oasis Ecology, Xinjiang Institute of Ecology and Geography, Chinese Academy of Sciences (G2018–02–07), the Fundamental Research Funds for the Central Universities (2572020DY17).

## Declaration of Competing Interest

The authors declare that they have no known competing financial interests or personal relationships which have, or could be perceived to have, influenced the work reported in this article.

## Appendix Supplementary materials

Image, application 1

Image, application 2

Image, application 3

Image, application 4

Image, application 5

Image, application 6

Image, application 7

## References

[1] Tseng E., Underwood J G (2013). Full length cDNA sequencing on the PacBio RS[J]. J biomolecular techniques: JBT.

[2] Sequencing Jenny Gu.Isoform (2014). Unveiling the Complex landscape in eukaryotic transcriptome on the pacBio® RS II[J]. Pag Asia.

[3] Waterhouse R.M., Mathieu S., Simão Felipe A. (2018). BUSCO Applications from Quality Assessments to Gene Prediction and Phylogenomics[J]. Molecular Biology & Evolution.

[4] Mathieu Seppey, Mosè (2019). BUSCO: assessing Genome Assembly and Annotation completeness[J]. Methods in Molecular Biology.

[5] Deng Y.Y., Li J.Q., Wu S.F. (2006). Integrated nr database in protein annotation system and its localization[J]. Computer Engineering.

[6] Ashburner M., Ball C.A., Blake J.A. (2000). Gene Ontology: tool for the unification of biology[J]. Nat. Genet..

[7] Tatusov R.L., Galperin M.Y., Natale D.A. (2000). The COG database: a tool for genome-scale analysis of protein functions and evolution[J]. Nuclc Acids Research.

[8] Huerta-Cepas J., Szklarczyk D., Forslund K. (2016). eggNOG 4.5: a hierarchical orthology framework with improved functional annotations for eukaryotic, prokaryotic and viral sequences[J]. Nucleic Acids Res..

[9] Apweiler R., Bairoch A., Wu C.H. (2004). UniProt: the universal protein knowledgebase[J]. Nucleic Acids Res..

[10] Haas B.J., Papanicolaou A., Yassour M. (2013). De novo transcript sequence reconstruction from RNA-Seq using the Trinity platform for reference generation and analysis[J]. Nature Protocol.

[11] Kanehisa M., Goto S., Kawashima S. (2004). The KEGG resource for deciphering the genome[J]. Nucleic Acids Res..

